# Asymptomatic Incidental Pyogenic Hepatic Abscess in an Obese Adult

**DOI:** 10.7759/cureus.17626

**Published:** 2021-08-31

**Authors:** Gagandeep S Samra, Johnny S Randhawa, Dipesh Patel, Roza Sabri

**Affiliations:** 1 Family Medicine, St. George's University School of Medicine, True Blue, GRD; 2 Family Medicine, Hackensack Meridian Health JFK University Medical Center, Edison, USA

**Keywords:** pyogenic hepatic abscess, group f streptococcus, streptococcus constellatus, incidental hepatic abscess, obese patient

## Abstract

A 35-year-old obese female patient presented to the emergency department (ED) endorsing symptoms of generalized weakness, dyspnea, and myalgia. Vitals on admission revealed hypotension, tachycardia, and a low-grade fever. Physical examination was unremarkable and was negative for any upper right quadrant tenderness or jaundice. Laboratory results revealed an elevated leukocytosis with a predominantly elevated neutrophil count, an elevated lactate dehydrogenase, aminotransferases, and an elevated anion gap. Sepsis protocol was initiated. Blood cultures revealed Group F Streptococcus. A chest x-ray for localization of the primary infection source was significant for an incidental hypodense liver mass. A follow-up magnetic resonance imaging (MRI) without contrast revealed a left hepatic multiloculated enhancing lesion prompting the diagnosis of beta-hemolytic Group F Streptococcus pyogenic hepatic abscess (PHA). This unusual case seeks to inform that an obese patient (i.e., immunocompromised) with systemic signs (e.g., fever, hypotension, tachycardia) should warrant careful monitoring as well as the inclusion of pyogenic liver abscess in the differential workup as our patient’s PHA was found incidentally on a chest x-ray. Appropriate management via sonographic guided drainage was initiated and systemic antibiotics were administered in both inpatient and outpatient settings, resulting in complete resolution of the hepatic abscess over the course of a month.

## Introduction

A pyogenic hepatic abscess (PHA) most commonly presents with symptoms of fever, right upper quadrant (RUQ) pain, and jaundice. Common risk factors include, but are not limited to, intravenous drug use, diabetes mellitus (DM), proton pump inhibitor use, and more [1]. PHAs are a rare entity in the USA with an occurrence rate of about 3.6 in every 100,000 people [1]. Other risk factors for PHA include male sex, advancing age, blunt trauma, malignancy, inflammatory bowel disease, diverticulitis, cirrhosis, appendicitis, perforated bowel, dental extraction, acupuncture, and hemorrhoidectomy [1-4]. The most common bacterial agents of PHA include gram-negative bacilli microbes such as Klebsiella, Escherichia coli, and Enterococcus [5]. A much lesser-known cause is Streptococcus constellatus, gram-positive, catalase-negative cocci, which is a normal commensal of the oropharynx, gastrointestinal and genitourinary systems [2,6,7]. In this report, we present a 35-year-old obese woman who arrived at the emergency department with sepsis and was found to have a concurrent asymptomatic PHA.

## Case presentation

**Figure 1:**
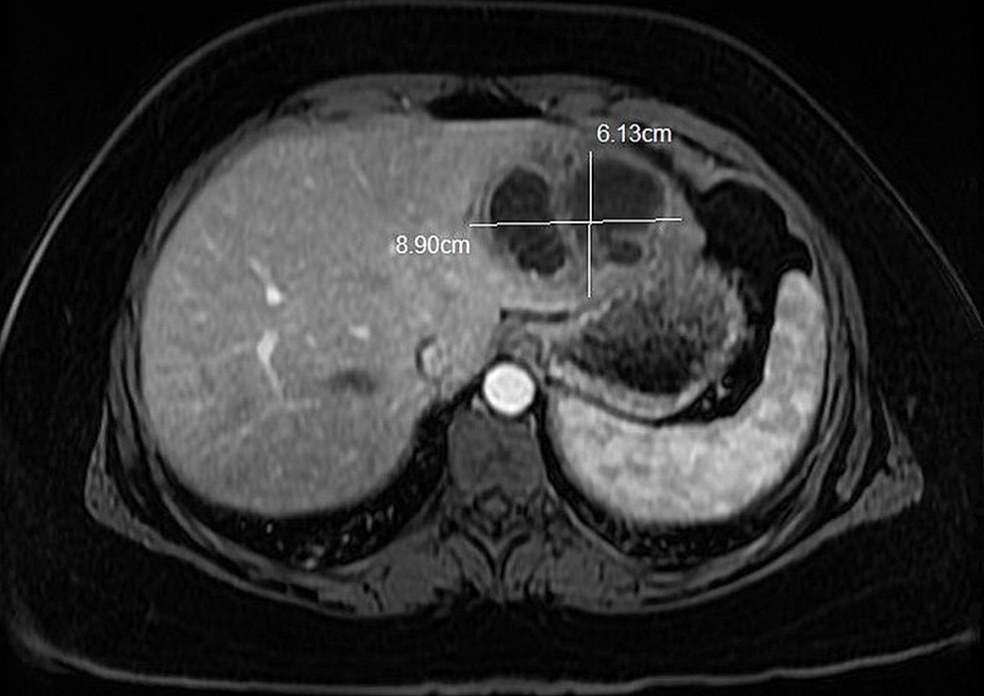
T-1 MRI abdomen with an annotated hepatic abscess in the lower left lobe

A 35-year-old Caucasian female with a medical history of obesity (body mass index of 39 kg/m^2^) with no travel history and not on any medications presented to the ED with symptoms of generalized weakness, body aches, and difficulty in breathing. Vital signs recorded blood pressure of 84/55 mmHg, a heart rate of 128 beats per minute, temperature of 100.6℉ (38.1℃), respiration rate of 18 breaths per minute, and oxygen saturation of 98%. Initial laboratory showed an elevated leukocytosis of 19.1 x 10^3^/µL (normal range: 4-11 x 10^3^/µL) with a predominant neutrophil count (ANC) of 17.6 x 10^3^/µL (normal range: 2-7.8 x 10^3^/µL). The patient’s lactic acid was elevated to 4.1 mmol/L (normal range: 0.5-2.2 mmol/L), as well as her alanine aminotransferase level (ALT) of 71 U/L (normal range: 8-38 U/L), aspartate aminoransferase (AST) level of 56 U/L (normal range: 12-33.9 U/L), and an anion gap of 24 mmol/L (normal range: 10-20 mmol/L). A portable chest x-ray (CXR) was performed and revealed pulmonary vascular congestion. Three out of the four systemic inflammatory response syndrome (SIRS) criteria were met leading to the initiation of the sepsis fluid protocol. Intravenous (IV) norepinephrine 5 mcg/min was also started for additional blood pressure support. Repeat labs revealed an up-trending leukocytosis to 22.5 x 10^3^/µL. The patient was being treated for sepsis pending work-up and started on IV vancomycin and IV piperacillin/tazobactam for broad-spectrum coverage as per infectious disease (ID). After stabilization, the patient was admitted to the intensive care unit (ICU) for further management. Results from the blood cultures showed Group F Streptococcus with sensitivity to ampicillin, ceftriaxone, penicillin, clindamycin, cefotaxime, and vancomycin. The antibiotic regimen was then narrowed to IV ceftriaxone 2g daily. A repeat CXR done on hospital day 7 revealed a hypodense liver mass. Further workup via magnetic resonance imaging (MRI) without contrast on day 8 revealed a left hepatic multiloculated enhancing lesion measuring 8.9 cm x 6.1 cm x 5.1 cm (Figure 1), consistent with a hepatic abscess and one 2 mm right hepatic cyst.

**Figure 2:**
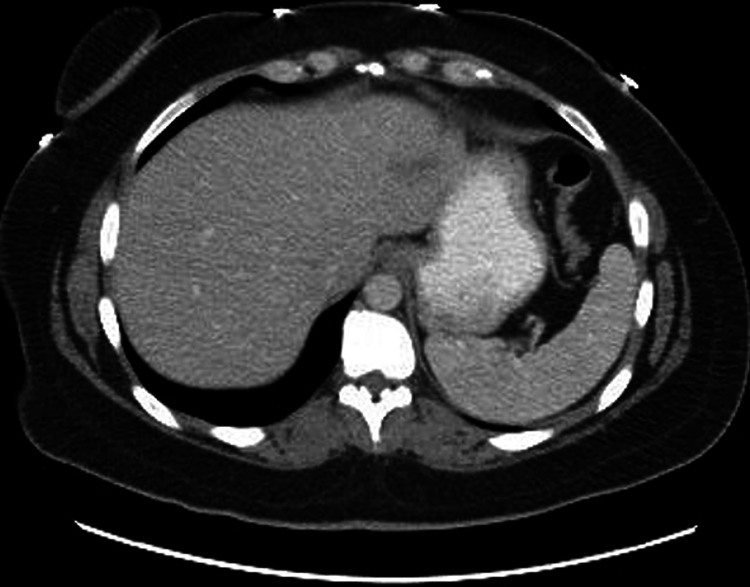
CT abdomen with resolving hepatic abscess in the left lower lobe

Gastrointestinal and Surgery specialties were consulted at this time and sonographic-guided drainage of the abscess was recommended. The following day, Interventional Radiology drained 15 mL of hemorrhagic purulent fluid with the placement of a Jackson-Pratt Drain (JP Drain). However, status post drainage the patient developed rigors and a maximum fever of 101℉ (38.3℃) and a rapid response was called until the patient was stabilized. Liver abscess cultures were negative for Entamoeba histolytica, ova, and parasites but positive for Group F Streptococcus constellatus. Per os (PO) metronidazole 500 mg was then added to the patient’s antibiotic regimen. Repeat computerized tomography (CT) scan with contrast on day 17 revealed a mild decrease in liver abscess size from 7.1 cm x 6.6 cm to 5.8 cm x 4.0 cm. As per surgery recommendations, the drain was kept in place and a CT scan was scheduled in two weeks. On day 19, WBC levels were down trended, and the patient was discharged on home infusion antibiotics via a peripherally inserted central catheter (PICC) line on a regimen of IV cefepime 2 g every eight hours, IV vancomycin 1g every 24 hours, and PO metronidazole 500 mg every eight hours for a minimum of three weeks as per ID recommendation. However, on the same evening after discharge, the patient reported feeling chills and returned to the ED. She was found to have a maximum temperature of 102.7℉ (39.2℃) and a WBC level of 23.1 10^3^/µL was noted on initial bloodwork. JP Drain disruption from mechanical pressure was ruled to be the cause of this transient decompensation. The patient was restabilized and WBC levels were down trended again until readmission day 12 when she was discharged on the previously planned antibiotic regimen. Outpatient follow-up one-week after discharge showed the patient in an uneventful state and on course to full recovery. A follow-up CT of the abdomen and pelvis with contrast one-month status post-discharge noted decreased liver abscess size to 3 cm, and the antibiotic regimen was changed to IV ceftriaxone and PO metronidazole as per ID recommendations. Further, follow-up imaging with a CT of the abdomen one month later noted a continued decrease in the size of the abscess to 2 cm (Figure 2). With the continued improvement noted on imaging, the antibiotic regimen was switched to PO cefdinir until complete resolution of the abscess was eventually achieved.

## Discussion

As described in previous literature, the risk of PHA development is increased with underlying risk factors such as IV drug abuse, DM, malignancy, other comorbidities [1]. Furthermore, there are often typical clinical signs and symptoms associated with PHA that can help guide clinicians toward accurate diagnosis and treatment including but not limited to fevers, chills, RUQ pain, high ALT, and high alkaline phosphatase concentrations [6,8]. However, our patient’s only co-morbidity was obesity, which can alter immune function [9]. She had lacked all potential risk factors for developing PHA as well as the typical physical symptoms that are commonly associated with PHAs. Due to this, it was clinically challenging to determine if the hepatic abscess caused the patient's bacteremia or if the bacteremia resulted in asymptomatic hepatic seeding. We suspect our patient to have had the asymptomatic abscess initially that resulted in spread to the blood, as hepatic seeding secondary from bacteremia would most commonly result in multiple abscesses [6]. Nonetheless, it is important to include normal body commensals as potential causative agents in the development of PHAs and bacteremia, particularly Group F Streptococcus constellatus [10]. As seen in this unique case, our patient only presented with symptoms of sepsis including non-specific symptoms of fever, hypotension, leukocytosis, generalized weakness, and body aches. Even without common classic symptoms of RUQ pain and jaundice, clinicians should not rule out the diagnosis of PHA in an obese patient.

## Conclusions

We present a unique case of an asymptomatic incidental PHA in a patient with obesity as their only co-morbidity. We aim to enlighten clinicians on such a case to ensure a comprehensive differential workup is done to rule out PHA even with a lack of typical hepatic abscess symptoms such as RUQ pain, fever, and jaundice.
